# (*E*)-1-(2-Fur­yl)-3-(2,4,6-trimeth­oxy­phen­yl)prop-2-en-1-one

**DOI:** 10.1107/S1600536810035762

**Published:** 2010-09-15

**Authors:** Hoong-Kun Fun, Thitipone Suwunwong, Suchada Chantrapromma, Chatchanok Karalai

**Affiliations:** a X-ray Crystallography Unit, School of Physics, Universiti Sains Malaysia, 11800 USM, Penang, Malaysia; b Crystal Materials Research Unit, Department of Chemistry, Faculty of Science, Prince of Songkla University, Hat-Yai, Songkhla 90112, Thailand

## Abstract

In the title heteroaryl chalcone derivative, C_16_H_16_O_5_, the dihedral angle between the furan and benzene rings is 14.45 (6)°. The three meth­oxy groups are almost coplanar with their attached benzene ring [C—C—O—C torsion angles = 2.07 (17), −5.04 (17) and 2.85 (16)°]. An intra­molecular C—H⋯O hydrogen bond occurs. In the crystal, adjacent mol­ecules are linked into X-shaped chains along the *c* axis by weak C—H⋯O(enone) inter­actions. These chains are stacked along the *b* axis. C⋯O [3.3308 (13)–3.4123 (14) Å] short contacts are also observed.

## Related literature

For bond-length data, see: Allen *et al.* (1987 ▶). For hydrogen-bond motifs, see: Bernstein *et al.* (1995 ▶). For related structures, see: Chantrapromma *et al.* (2009 ▶); Suwunwong *et al.* (2009 ▶. For background to and applications of chalcones and heteroaryl chalcones, see: Gaber *et al.* (2008 ▶); Go *et al.* (2005 ▶); Jung *et al.* (2008 ▶); Ng *et al.* (2009 ▶); Ni *et al.* (2004 ▶); Nowakowska (2007 ▶); Patil & Dharmaprakash (2008 ▶) and Tewtrakul *et al.* (2003 ▶). For the stability of the temperature controller used in the data collection, see Cosier & Glazer, (1986 ▶).
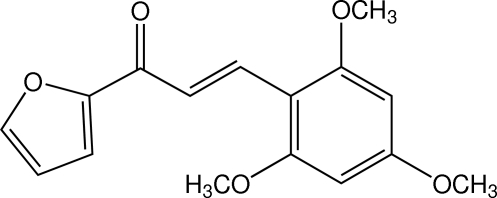


## Experimental

### 

#### Crystal data

C_16_H_16_O_5_
*M* *_r_* = 288.29
Monoclinic, 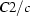

*a* = 38.5688 (5) Å
*b* = 3.93493 (5) Å
*c* = 18.2638 (3) Å
β = 103.901 (1)°
*V* = 2690.68 (6) Å^3^
*Z* = 8
Mo *K*α radiation
μ = 0.11 mm^−1^
*T* = 100 K
0.41 × 0.15 × 0.09 mm

#### Data collection

Bruker APEXII CCD diffractometer
Absorption correction: multi-scan (*SADABS*; Bruker, 2005 ▶) *T*_min_ = 0.957, *T*_max_ = 0.990
20657 measured reflections
3941 independent reflections
3077 reflections with *I* > 2σ(*I*)
*R*_int_ = 0.038

#### Refinement

*R*[*F* ^2^ > 2σ(*F* ^2^)] = 0.041
*wR*(*F* ^2^) = 0.113
*S* = 1.06
3941 reflections
254 parameters
All H-atom parameters refined
Δρ_max_ = 0.35 e Å^−3^
Δρ_min_ = −0.24 e Å^−3^

### 

Data collection: *APEX2* (Bruker, 2005 ▶); cell refinement: *SAINT* (Bruker, 2005 ▶); data reduction: *SAINT*; program(s) used to solve structure: *SHELXTL* (Sheldrick, 2008 ▶); program(s) used to refine structure: *SHELXTL*; molecular graphics: *SHELXTL*; software used to prepare material for publication: *SHELXTL* and *PLATON* (Spek, 2009 ▶).

## Supplementary Material

Crystal structure: contains datablocks global, I. DOI: 10.1107/S1600536810035762/hb5630sup1.cif

Structure factors: contains datablocks I. DOI: 10.1107/S1600536810035762/hb5630Isup2.hkl

Additional supplementary materials:  crystallographic information; 3D view; checkCIF report

## Figures and Tables

**Table 1:** Hydrogen-bond geometry (Å, °)

*D*—H⋯*A*	*D*—H	H⋯*A*	*D*⋯*A*	*D*—H⋯*A*
C1—H1⋯O2^i^	0.956 (15)	2.496 (15)	3.3512 (14)	148.9 (13)
C6—H6⋯O5	0.965 (15)	2.260 (14)	2.8197 (15)	116.0 (11)
C14—H14*A*⋯O4^ii^	0.975 (15)	2.589 (16)	3.4462 (14)	146.7 (11)
C15—H15*A*⋯O1^iii^	0.989 (16)	2.546 (16)	3.4293 (18)	148.6 (12)
C16—H16*A*⋯O1^iii^	0.982 (16)	2.575 (16)	3.4120 (16)	143.0 (12)

Symmetry codes: (i) 
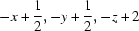
; (ii) 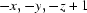; (iii) 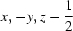.

**Fig. 1.**
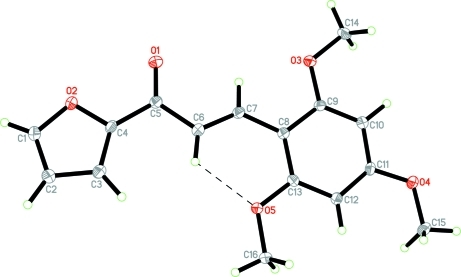
The molecular structure of (I), showing 50% probability displacement ellipsoids. Weak intramolecular interactions are shown as dashed lines.

**Fig. 2.**
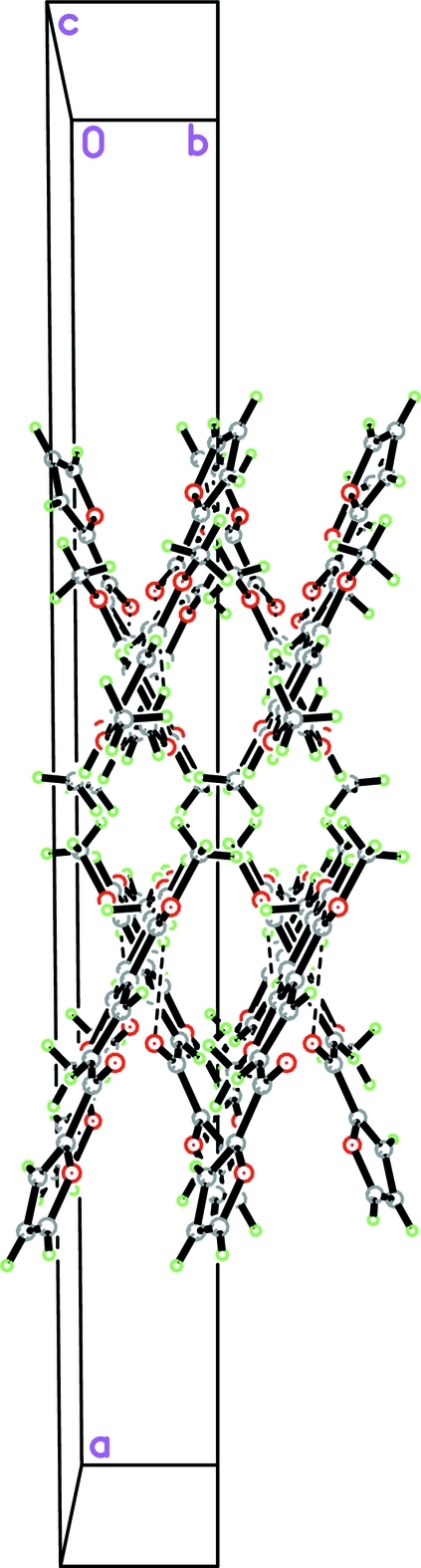
The crystal packing of (I) viewed along the c axis, showing X-chains running along the c axis. Weak C—H···O interactions are shown as dashed lines.

**Fig. 3.**
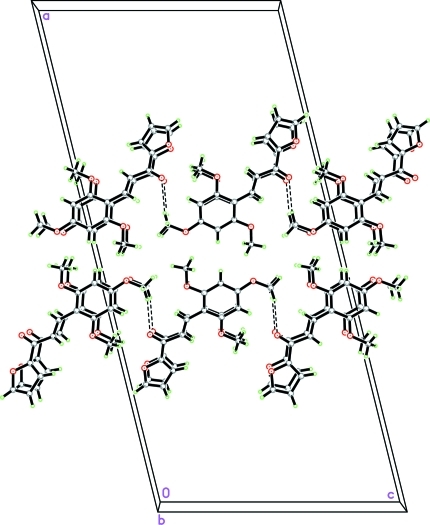
The crystal packing of (I) viewed along the b axis, showing chains stacking along the b axis. Weak C—H···O interactions are shown as dashed lines.

## supplementary crystallographic information

### Comment

Chalcone and heteroaryl chalcones are very interesting due to their variety of applications with biological activities. Many of them possess analgesic, anti-inflammatory and antibacterial properties (Go *et al.*, 2005; Ni *et al.*, 2004; Nowakowska, 2007) as well as HIV-1 protease inhibitory (Tewtrakul *et al.*, 2003) and tyrosinase inhibitory (Ng *et al.*, 2009) activities. Moreover synthetic chalcones and heteroaryl chalcones have also been found to exhibit non-linear optical (Patil & Dharmaprakash, 2008), fluorescent (Jung *et al.*, 2008) and laser properties (Gaber *et al.*, 2008) . In continuing our on-going research on antibacterial activities and fluorescence properties of chalcones and heteroaryl chalcone derivatives, the title heteroaryl chalcone was synthesized in order to study its antibacterial and fluorescence properties. However our results show that (I) do not possess fluorescence property. In addition our biological testing found that (I) was inactive against the tested bacteria strains which are *Bacillus subtilis*, *Enterococcus faecalis*, *Staphylococcus aureus*, Methicillin-Resistant *Staphylococcus aureus*, Vancomycin-Resistant *Enterococcus faecalis*, *Pseudomonas aeruginosa*, *Salmonella typhi* and *Shigella sonnei*. Herein we report the crystal structure of (I).

The molecule of the title heteroaryl chalcone (Fig. 1) exists in an *E* configuration with respect to the C6═C7 double bond [1.3512 (16) Å] with the C5–C6–C7–C8 torsion angle being -176.44 (12)°. The whole molecule is slightly twisted with the dihedral angle between the furan and benzene rings being 14.45 (6)°. Atoms of the propenone unit (C5, C6, C7 and O1) lie on the same plane [*r.m.s*. 0.0179 (1)]. This plane makes dihedral angles of 11.38 (8) and 9.12 (8)° with furan and phenyl rings, respectively. All the three substituted methoxy groups of 2,4,6-trimethoxyphenyl unit are almost co-planar with the phenyl ring as indicated by torsion angles C14–O3–C9–C10 = 2.07 (17)°, C15–O4–C11–C12 = -5.04 (17)° and C16–O5–C13–C12 =  2.85 (16)°. In the structure, a weak intramolecular C6—H6···O5  interaction generates an S(6) ring motif (Bernstein *et al.*, 1995) (Table 1). The bond lengths have normal values (Allen *et al.*, 1987) and bond lengths and angles are comparable with its related structures (Chantrapromma *et al.*, 2009; Suwunwong *et al.*, 2009).

In the crystal packing, all the three methoxy groups involve in weak intermolecular C—H···O interactions (Table 1). The adjacent molecules are linked into X-shape chains along the *c* axis through the enone unit by weak C—H···O interactions (Fig. 2, Table 1). The adjacent chains are arranged into face-to-face manner (Fig. 3) and stacked along the *b* axis (Fig. 3). The crystal is further stabilized by C···O[3.3308 (13)-3.4123 (14) Å] short contacts.

### Experimental

The title compound was prepared by the condensation of the solution of 2-furyl methylketone (2 mmol, 0.22 g) in ethanol (15 ml) and 2,4,6-trimethoxybenzaldehyde (2 mmol, 0.40 g) in ethanol (15 ml) in the presence of 20% NaOH (aq) 5 ml at 278 K for 5 hr. The resulting solid which was obtained was further collected by filtration, washed with distilled water and dried in air. Colorless blocks of (I) were recrystalized from acetone/ethanol (1:1 *v*/*v*) by the slow evaporation of the solvent at room temperature after several days, Mp. 390–391 K.

### Refinement

All H atoms were located in difference maps and refined isotropically. The highest residual electron density peak is located at 0.63 Å from C10 and the deepest hole is located at 1.12 Å from C2.

### Crystal data

**Table:**

C_16_H_16_O_5_	*F*(000) = 1216
*M**_r_* = 288.29	*D*_x_ = 1.423 Mg m^−^^3^
Monoclinic, *C*2/*c*	Melting point = 390–391 K
Hall symbol: -C 2yc	Mo *K*α radiation, λ = 0.71073 Å
*a* = 38.5688 (5) Å	Cell parameters from 3941 reflections
*b* = 3.93493 (5) Å	θ = 1.1–30.0°
*c* = 18.2638 (3) Å	µ = 0.11 mm^−^^1^
β = 103.901 (1)°	*T* = 100 K
*V* = 2690.68 (6) Å^3^	Block, colorless
*Z* = 8	0.41 × 0.15 × 0.09 mm

### Data collection

**Table:**

Bruker APEXII CCD diffractometer	3941 independent reflections
Radiation source: sealed tube	3077 reflections with *I* > 2σ(*I*)
graphite	*R*_int_ = 0.038
φ and ω scans	θ_max_ = 30.0°, θ_min_ = 1.1°
Absorption correction: multi-scan (*SADABS*; Bruker, 2005)	*h* = −54→54
*T*_min_ = 0.957, *T*_max_ = 0.990	*k* = −5→5
20657 measured reflections	*l* = −25→25

### Refinement

**Table:**

Refinement on *F*^2^	Primary atom site location: structure-invariant direct methods
Least-squares matrix: full	Secondary atom site location: difference Fourier map
*R*[*F*^2^ > 2σ(*F*^2^)] = 0.041	Hydrogen site location: inferred from neighbouring sites
*wR*(*F*^2^) = 0.113	All H-atom parameters refined
*S* = 1.06	*w* = 1/[σ^2^(*F*_o_^2^) + (0.0556*P*)^2^ + 1.4768*P*] where *P* = (*F*_o_^2^ + 2*F*_c_^2^)/3
3941 reflections	(Δ/σ)_max_ = 0.001
254 parameters	Δρ_max_ = 0.35 e Å^−^^3^
0 restraints	Δρ_min_ = −0.24 e Å^−^^3^

### Special details

**Table:**

Experimental. The crystal was placed in the cold stream of an Oxford Cryosystems Cobra open-flow nitrogen cryostat (Cosier & Glazer, 1986) operating at 120.0 (1) K.
Geometry. All esds (except the esd in the dihedral angle between two l.s. planes) are estimated using the full covariance matrix. The cell esds are taken into account individually in the estimation of esds in distances, angles and torsion angles; correlations between esds in cell parameters are only used when they are defined by crystal symmetry. An approximate (isotropic) treatment of cell esds is used for estimating esds involving l.s. planes.
Refinement. Refinement of F^2^ against ALL reflections. The weighted R-factor wR and goodness of fit S are based on F^2^, conventional R-factors R are based on F, with F set to zero for negative F^2^. The threshold expression of F^2^ > 2sigma(F^2^) is used only for calculating R-factors(gt) etc. and is not relevant to the choice of reflections for refinement. R-factors based on F^2^ are statistically about twice as large as those based on F, and R- factors based on ALL data will be even larger.

### Fractional atomic coordinates and isotropic or equivalent isotropic displacement parameters (Å^2^)

**Table:**

	*x*	*y*	*z*	*U*_iso_*/*U*_eq_	
O1	0.15077 (2)	0.0988 (3)	0.84899 (5)	0.0215 (2)	
O2	0.21727 (2)	0.3478 (2)	0.90839 (4)	0.01663 (19)	
O3	0.05690 (2)	−0.2197 (2)	0.65467 (5)	0.01694 (19)	
O4	0.04708 (2)	−0.1724 (2)	0.39117 (5)	0.01735 (19)	
O5	0.15024 (2)	0.2851 (2)	0.56781 (5)	0.01639 (19)	
C1	0.25039 (3)	0.4913 (3)	0.92049 (7)	0.0174 (2)	
H1	0.2651 (4)	0.474 (4)	0.9706 (8)	0.018 (4)*	
C2	0.25591 (3)	0.6274 (3)	0.85645 (7)	0.0188 (3)	
H2	0.2775 (4)	0.741 (4)	0.8513 (9)	0.027 (4)*	
C3	0.22403 (3)	0.5659 (3)	0.79963 (7)	0.0174 (2)	
H3	0.2194 (4)	0.630 (4)	0.7461 (9)	0.021 (4)*	
C4	0.20144 (3)	0.3929 (3)	0.83315 (6)	0.0141 (2)	
C5	0.16581 (3)	0.2391 (3)	0.80482 (6)	0.0150 (2)	
C6	0.15171 (3)	0.2521 (3)	0.72275 (7)	0.0153 (2)	
H6	0.1649 (4)	0.381 (4)	0.6935 (8)	0.019 (4)*	
C7	0.12181 (3)	0.0762 (3)	0.69077 (6)	0.0145 (2)	
H7	0.1101 (4)	−0.049 (4)	0.7240 (8)	0.020 (4)*	
C8	0.10376 (3)	0.0332 (3)	0.61194 (6)	0.0133 (2)	
C9	0.06997 (3)	−0.1318 (3)	0.59416 (6)	0.0132 (2)	
C10	0.05156 (3)	−0.1931 (3)	0.52043 (7)	0.0150 (2)	
H10	0.0282 (4)	−0.310 (4)	0.5082 (9)	0.027 (4)*	
C11	0.06682 (3)	−0.0939 (3)	0.46185 (6)	0.0140 (2)	
C12	0.09968 (3)	0.0689 (3)	0.47557 (6)	0.0139 (2)	
H12	0.1100 (4)	0.127 (4)	0.4359 (8)	0.018 (4)*	
C13	0.11766 (3)	0.1321 (3)	0.55034 (6)	0.0131 (2)	
C14	0.02326 (3)	−0.3920 (3)	0.63942 (7)	0.0177 (2)	
H14A	0.0040 (4)	−0.255 (4)	0.6091 (8)	0.018 (4)*	
H14B	0.0253 (4)	−0.612 (4)	0.6129 (8)	0.021 (4)*	
H14C	0.0183 (4)	−0.431 (4)	0.6887 (9)	0.023 (4)*	
C15	0.06015 (4)	−0.0546 (4)	0.32866 (7)	0.0206 (3)	
H15A	0.0833 (4)	−0.158 (4)	0.3269 (9)	0.024 (4)*	
H15B	0.0411 (5)	−0.122 (4)	0.2834 (9)	0.032 (4)*	
H15C	0.0620 (4)	0.196 (4)	0.3296 (9)	0.024 (4)*	
C16	0.16511 (3)	0.3953 (3)	0.50694 (7)	0.0158 (2)	
H16A	0.1703 (4)	0.200 (4)	0.4778 (9)	0.021 (4)*	
H16B	0.1490 (4)	0.555 (4)	0.4762 (8)	0.015 (3)*	
H16C	0.1875 (4)	0.511 (4)	0.5307 (8)	0.020 (4)*	

### Atomic displacement parameters (Å^2^)

**Table:**

	*U*^11^	*U*^22^	*U*^33^	*U*^12^	*U*^13^	*U*^23^
O1	0.0194 (4)	0.0314 (5)	0.0145 (4)	−0.0052 (4)	0.0057 (3)	0.0020 (4)
O2	0.0151 (4)	0.0227 (5)	0.0111 (4)	−0.0018 (3)	0.0012 (3)	0.0000 (3)
O3	0.0143 (4)	0.0231 (5)	0.0142 (4)	−0.0047 (3)	0.0049 (3)	0.0000 (3)
O4	0.0162 (4)	0.0239 (5)	0.0111 (4)	−0.0038 (3)	0.0015 (3)	−0.0018 (3)
O5	0.0130 (4)	0.0242 (5)	0.0119 (4)	−0.0051 (3)	0.0029 (3)	0.0009 (3)
C1	0.0152 (5)	0.0204 (6)	0.0157 (6)	−0.0013 (4)	0.0017 (4)	−0.0018 (5)
C2	0.0174 (6)	0.0211 (6)	0.0182 (6)	−0.0039 (5)	0.0049 (5)	−0.0022 (5)
C3	0.0193 (6)	0.0193 (6)	0.0133 (5)	−0.0018 (4)	0.0037 (4)	−0.0014 (5)
C4	0.0152 (5)	0.0171 (6)	0.0094 (5)	0.0016 (4)	0.0016 (4)	−0.0009 (4)
C5	0.0149 (5)	0.0171 (6)	0.0131 (5)	0.0011 (4)	0.0038 (4)	−0.0007 (4)
C6	0.0155 (5)	0.0172 (6)	0.0131 (5)	−0.0002 (4)	0.0033 (4)	0.0004 (4)
C7	0.0146 (5)	0.0169 (6)	0.0122 (5)	0.0018 (4)	0.0038 (4)	−0.0003 (4)
C8	0.0129 (5)	0.0147 (5)	0.0124 (5)	0.0009 (4)	0.0032 (4)	−0.0001 (4)
C9	0.0135 (5)	0.0139 (5)	0.0129 (5)	0.0006 (4)	0.0048 (4)	0.0008 (4)
C10	0.0125 (5)	0.0170 (6)	0.0150 (5)	−0.0007 (4)	0.0027 (4)	−0.0006 (4)
C11	0.0132 (5)	0.0153 (6)	0.0121 (5)	0.0011 (4)	0.0003 (4)	−0.0012 (4)
C12	0.0133 (5)	0.0163 (6)	0.0121 (5)	0.0014 (4)	0.0031 (4)	0.0006 (4)
C13	0.0106 (5)	0.0133 (5)	0.0152 (5)	−0.0001 (4)	0.0028 (4)	0.0002 (4)
C14	0.0142 (5)	0.0197 (6)	0.0199 (6)	−0.0029 (4)	0.0058 (5)	0.0018 (5)
C15	0.0232 (6)	0.0265 (7)	0.0117 (5)	−0.0043 (5)	0.0036 (5)	0.0000 (5)
C16	0.0143 (5)	0.0205 (6)	0.0133 (5)	−0.0023 (4)	0.0043 (4)	0.0024 (5)

### Geometric parameters (Å, °)

**Table:**

O1—C5	1.2315 (14)	C7—C8	1.4507 (16)
O2—C1	1.3652 (14)	C7—H7	0.974 (15)
O2—C4	1.3745 (13)	C8—C13	1.4123 (15)
O3—C9	1.3648 (13)	C8—C9	1.4220 (15)
O3—C14	1.4307 (14)	C9—C10	1.3844 (16)
O4—C11	1.3673 (14)	C10—C11	1.3949 (16)
O4—C15	1.4317 (15)	C10—H10	0.988 (16)
O5—C13	1.3602 (13)	C11—C12	1.3883 (16)
O5—C16	1.4352 (14)	C12—C13	1.3974 (16)
C1—C2	1.3492 (17)	C12—H12	0.936 (15)
C1—H1	0.956 (15)	C14—H14A	0.976 (15)
C2—C3	1.4264 (17)	C14—H14B	1.003 (16)
C2—H2	0.970 (16)	C14—H14C	0.977 (16)
C3—C4	1.3617 (17)	C15—H15A	0.989 (16)
C3—H3	0.984 (15)	C15—H15B	1.001 (17)
C4—C5	1.4766 (16)	C15—H15C	0.990 (17)
C5—C6	1.4675 (16)	C16—H16A	0.982 (16)
C6—C7	1.3512 (16)	C16—H16B	0.963 (15)
C6—H6	0.967 (15)	C16—H16C	0.982 (16)
			
C1—O2—C4	106.39 (9)	C9—C10—C11	119.01 (10)
C9—O3—C14	117.18 (9)	C9—C10—H10	121.8 (9)
C11—O4—C15	117.18 (9)	C11—C10—H10	119.2 (9)
C13—O5—C16	118.08 (9)	O4—C11—C12	123.50 (10)
C2—C1—O2	111.12 (10)	O4—C11—C10	114.76 (10)
C2—C1—H1	132.6 (9)	C12—C11—C10	121.74 (10)
O2—C1—H1	116.2 (9)	C11—C12—C13	118.39 (10)
C1—C2—C3	105.98 (11)	C11—C12—H12	120.9 (9)
C1—C2—H2	125.8 (10)	C13—C12—H12	120.7 (9)
C3—C2—H2	128.2 (10)	O5—C13—C12	121.43 (10)
C4—C3—C2	106.90 (11)	O5—C13—C8	116.18 (10)
C4—C3—H3	126.4 (9)	C12—C13—C8	122.37 (10)
C2—C3—H3	126.7 (9)	O3—C14—H14A	112.3 (9)
C3—C4—O2	109.60 (10)	O3—C14—H14B	109.3 (8)
C3—C4—C5	133.59 (11)	H14A—C14—H14B	109.8 (12)
O2—C4—C5	116.74 (10)	O3—C14—H14C	105.3 (9)
O1—C5—C6	124.57 (11)	H14A—C14—H14C	108.5 (12)
O1—C5—C4	119.95 (10)	H14B—C14—H14C	111.5 (13)
C6—C5—C4	115.40 (10)	O4—C15—H15A	112.7 (9)
C7—C6—C5	119.48 (11)	O4—C15—H15B	104.0 (10)
C7—C6—H6	122.6 (9)	H15A—C15—H15B	110.8 (13)
C5—C6—H6	117.9 (9)	O4—C15—H15C	110.3 (9)
C6—C7—C8	130.17 (11)	H15A—C15—H15C	110.6 (13)
C6—C7—H7	117.8 (9)	H15B—C15—H15C	108.2 (14)
C8—C7—H7	112.0 (9)	O5—C16—H16A	110.8 (9)
C13—C8—C9	116.51 (10)	O5—C16—H16B	109.1 (8)
C13—C8—C7	125.09 (10)	H16A—C16—H16B	112.5 (12)
C9—C8—C7	118.36 (10)	O5—C16—H16C	105.8 (8)
O3—C9—C10	122.72 (10)	H16A—C16—H16C	109.3 (12)
O3—C9—C8	115.31 (10)	H16B—C16—H16C	109.2 (13)
C10—C9—C8	121.96 (10)		
			
C4—O2—C1—C2	0.63 (14)	C7—C8—C9—O3	3.17 (16)
O2—C1—C2—C3	−0.01 (15)	C13—C8—C9—C10	0.06 (17)
C1—C2—C3—C4	−0.63 (14)	C7—C8—C9—C10	−177.77 (11)
C2—C3—C4—O2	1.03 (14)	O3—C9—C10—C11	179.79 (11)
C2—C3—C4—C5	−175.73 (13)	C8—C9—C10—C11	0.80 (18)
C1—O2—C4—C3	−1.03 (13)	C15—O4—C11—C12	−5.04 (17)
C1—O2—C4—C5	176.35 (10)	C15—O4—C11—C10	175.53 (11)
C3—C4—C5—O1	−178.75 (13)	C9—C10—C11—O4	178.49 (10)
O2—C4—C5—O1	4.67 (17)	C9—C10—C11—C12	−0.95 (18)
C3—C4—C5—C6	4.4 (2)	O4—C11—C12—C13	−179.17 (11)
O2—C4—C5—C6	−172.18 (10)	C10—C11—C12—C13	0.22 (18)
O1—C5—C6—C7	−6.08 (19)	C16—O5—C13—C12	2.85 (16)
C4—C5—C6—C7	170.60 (11)	C16—O5—C13—C8	−178.91 (10)
C5—C6—C7—C8	−176.44 (12)	C11—C12—C13—O5	178.81 (10)
C6—C7—C8—C13	10.2 (2)	C11—C12—C13—C8	0.69 (18)
C6—C7—C8—C9	−172.18 (12)	C9—C8—C13—O5	−179.03 (10)
C14—O3—C9—C10	2.07 (17)	C7—C8—C13—O5	−1.37 (17)
C14—O3—C9—C8	−178.87 (10)	C9—C8—C13—C12	−0.82 (17)
C13—C8—C9—O3	−179.00 (10)	C7—C8—C13—C12	176.84 (11)

### Hydrogen-bond geometry (Å, °)

**Table:**

*D*—H···*A*	*D*—H	H···*A*	*D*···*A*	*D*—H···*A*
C1—H1···O2^i^	0.956 (15)	2.496 (15)	3.3512 (14)	148.9 (13)
C6—H6···O5	0.965 (15)	2.260 (14)	2.8197 (15)	116.0 (11)
C14—H14A···O4^ii^	0.975 (15)	2.589 (16)	3.4462 (14)	146.7 (11)
C15—H15A···O1^iii^	0.989 (16)	2.546 (16)	3.4293 (18)	148.6 (12)
C16—H16A···O1^iii^	0.982 (16)	2.575 (16)	3.4120 (16)	143.0 (12)

Symmetry codes: (i) −*x*+1/2, −*y*+1/2, −*z*+2;  (ii) −*x*, −*y*, −*z*+1; (iii) *x*, −*y*, *z*−1/2.
